# The Relationship Between the Implementation of Statutory Preventative Measures, Perceived Susceptibility of COVID-19, and Personality Traits in the Initial Stage of Corona-Related Lockdown: A German and Austrian Population Online Survey

**DOI:** 10.3389/fpsyt.2021.596281

**Published:** 2021-01-27

**Authors:** Christiane Eichenberg, Martin Grossfurthner, Jeannine Andrich, Lisa Hübner, Sybille Kietaibl, Stefana Holocher-Benetka

**Affiliations:** ^1^Faculty of Medicine, Institute of Psychosomatics, Sigmund Freud University, Vienna, Austria; ^2^Faculty of Psychology, Sigmund Freud University, Vienna, Austria; ^3^Faculty of Medicine, Sigmund Freud University, Vienna, Austria; ^4^Evangelical Hospital, Vienna, Austria

**Keywords:** COVID-19, lockdown, preventive measure, health belief model, online-survey

## Abstract

**Background:** Particularly during the early and middle stages of the COVID-19 pandemic, a population's compliance with precautionary measures (e.g., hygiene rules, smart working, travel restrictions, and quarantine) is paramount in preventing the virus from spreading.

**Objective:** The investigation and documentation of different socio-demographic and personality-specific factors in regards to preventative measures and consequent specific health behaviors during the COVID-19 pandemic, based on the Health Belief Model.

**Method:** An online survey was conducted on *N* = 3,006 individuals living in Germany and Austria during the early stages of lockdown. The questionnaire consisted of a self-administered section, exploring the dimensions posited in the Health Belief Model: perceived severity, perceived susceptibility, perceived barriers, perceived benefits of health-promoting measures, and engagement in health-promoting behaviors. Additionally, the following standardized scales were used to record personality determinants: the Stress Coping Style Questionnaire SVF 78 to evaluate coping and processing strategies in stressful circumstances, the Positive and Negative Affect Schedule (PANAS) to assess the emotional state induced by the coronavirus crisis, the UI-18 scale to diagnose the intolerance of uncertainty, and the State-Trait Anxiety Inventory (STAI) to assess anxiety.

**Results:** In line with the Health Belief model, four groups were created based on perceived susceptibility and engagement in health-promoting behaviors, and consequently studied in relation to personality determinants. Those four groups differed significantly in regards to almost all personality dimensions (*p* ≤ 0.005). Group 1 (*n* = 450) shows a reduced engagement with protective measures and displays underestimation of the COVID-19-pandemic. Group 2 (*n* = 984) displays many positive personality variables and high compliance with protective measures. Group 3 (*n* = 468) perceives the subjective risk of disease as high, but high emotional discomfort and stress caused by the protective measures leads to the activation of a complex fear defense. Group 4 (*n* = 1,004) is highly anxious and therefore compliant.

**Conclusion:** This typification has implications for establishing the appropriate support systems. This is particularly important to encourage compliance with preventive regulations within the groups, which showed poor abidance for several reasons. For Group 1, further education on the realistic threat and efficient protective measures is as central as the fostering of empathy for others; with its resource-conscious exemplary behavior Group 2 could be used as a positive social role model. Group 3 would benefit from promoting self-care, while Group 4 requires information on psychosocial assistance availability in order to mitigate the high stress to which the group members are subjected.

## Introduction

Along with the medical challenges of the disease, the uncertainty regarding its treatment, and the management of the healthcare system, the COVID-19 outbreak has had a substantial psychosocial impact on the world's population. COVID-19 has produced a plethora of concerns and fears across the world, affecting several aspects of life: health, unemployment, grief, loss, and socialization (1). Accordingly, studies have mainly been focused on the common psychological effects fueled by the pandemic in different countries [e.g., China: (2, 3); Spain: (4); Italy: (5)], specific stressors such as quarantine measures [e.g., (6)], the fear triggered by news reports (7), the development of suitable instruments for the measurement of psychosocial stress (8, 9), notable psychological responses such as anxiety (10) or the development of pandemic stress reactions within risk groups (11). Further studies have addressed the clinically relevant effects of the COVID-19 pandemic for specific target groups [e.g., PTSD for healthcare professionals: (12), Chinese students: (13) or the elderly: (14)], the influence of a lockdown on factors determining the quality of life such as sexual activity (15) and sleep (16) or the treatment of critical cohorts such as those dealing with anxiety (17) and people living with diabetes (18). Despite a large number of studies covering the psychological burden of the pandemic, cultural differences in stress responses, country-specific availability of assistance options, and a regional different disease evolution, call for an in-depth analysis of each affected country [e.g., (19)].

The Health Belief Model (HBM) is a theoretical model concerned with health decision-making, developed in the 1950s (20). The model aims to explain the conditions under which a person will or will not engage in health behaviors. The HBM has the advantage of specifying a discrete set of common sense cognitions that appear to mediate the effects of demographic variables. It has been applied to the prediction of an impressively broad range of health behaviors among a wide range of populations [c.f. (21), p. 32]. Janz and Becker reviewed the HBM literature in 1984 and listed preventive behaviors examined from an HBM perspective in the context of hypertension, smoking, alcohol use, dieting, and exercise, influenza vaccination, dental visits and attendance at blood pressure. In many subsequent studies the model has been used to develop successful health communication interventions by targeting messages at the HBM variables to change health behaviors [e.g., (22–25)]. The HBM originally consisted of four key concepts: perceived susceptibility (vulnerability), perceived severity, perceived benefits, and perceived barriers. Together, these four concepts were proposed to account for a person's readiness to engage in preventive action such as the implementation of statutory preventative measures. For instance, the perceived threat and cost-benefit analysis increase the likelihood of a health-related behavior change. The sense of threat stems from the perceived severity (“COVID-19 can be fatal”) and perceived susceptibility (“I have an increased risk of COVID-19 infection”). Ultimately, the cost-benefit analysis considers the following variables: perceived barriers (“Adhesion to the preventive measures causes the loss of personal freedom”) and perceived benefits (“Adhesion to the preventive measures decreases the risk of contracting COVID-19”). Further development of the model by Becker (26) proposed additional “modifying factors” such as sex, age, and social class. The results of quantitative reviews of the susceptibility, severity, benefit, and barrier constructs suggest that these variables are very often found to be significant predictors of health-related behaviors [c.f. (27, 28)], we, therefore, assume that the Health Belief Model is a suitable heuristic model explaining health-related behaviors during the critical situation of a pandemic. However, since the relationship between the assessment of the individual risk and the enforcement of preventive measures is complicated, further investigation is necessary. As Lippke and Renneberg (29) point out, the influence of socio-demographic and psychological variables is often overlooked.

We thus decided to expand the HBM for the purpose of this study, by incorporating approaches from trauma research. Since a pandemic of this severity creates an extreme situation, connecting these concepts seemed particularly promising. To our knowledge, this approach is novel. According to previous research on stress and psychotraumatology, different groups emerge in the context of psychosocial emergency care in era-defining crises (1). The current pandemic is no exception. While some groups respond well, others are more likely to report psychological, psychosomatic, and somatopsychic symptoms, due to specific socio-demographic, and other predisposing factors. We postulate that these predisposing factors influencing traumatic processing, are also essential moderators of the engagement in health-promoting measures. One of the predisposing factors is mental morbidity prior to trauma, leading to a very intense emotional reaction during the traumatic experience. The intensity of emotional reaction in turn contributes to later psychopathology. However, how a crisis is overcome, ultimately depends on the individual relation between protective factors and risk factors.

In line with this concept, we consider a further differentiation of the HBM. In the early phase of the pandemic, when stress or trauma-related issues are less likely to arise, populations can be grouped according to (non-)compliance with preventive measures as a result of socio-demographic and personality-specific characteristics. The Health Belief Model, as a heuristic method, explains, and predicts health-related behaviors and can be extended to socio-demographic and particular personality-related factors that reflect the premorbid mental vulnerability and the intensity of emotional reaction during the crisis. The latter is constituted through certain dominant emotions (e.g., fear and anger), increased worries over COVID-19, personality traits persistently associated with fear, individual strategies to cope with insecurity, and chronic stress processing mechanisms. A better understanding of the groups mentioned above will facilitate compliance with preventive measures as group-based behavior-change interventions achieve better results than general measures (30). What influences compliance with both voluntary and statutory precautionary measures in the context of pandemics is currently unknown. As a detailed study of the benefits of integrative models is lacking [e.g., (31)], this study represents a significant contribution.

In the line with the Health Belief Model's prediction of health behaviors, this study aims to document and examine socio-demographic differences in compliance with preventive measures during the COVID-19 pandemic. Furthermore, it explores whether personality-related factors can serve as grouping criteria.

## Materials and Methods

### Study Design

The Austrian and German population were invited to participate in an online survey via social media (e.g., Facebook), online forums (home, gardening, cooking, video games, education), and newspapers. The online survey software SoSci Survey (https://www.soscisurvey.de) was used for data collection. A pre-test with ten participants was conducted and allowed for improvement in terms of the survey's feasibility, intelligibility, and comprehensiveness. The survey was available online from March 22–29, (start of lock-down in Austria: March 16, 2020; start of lock-down in Germany: March 23, 2020). Participants received information about the study design and data protection before filling in the questionnaire. The duration of the questionnaire was ~25 min. The Ethics Commission of Sigmund Freud University Vienna approved this study (date of approval: March 18, 2020).

### Instruments

Participants completed a self-administered questionnaire including socio-demographic data and various sets of questions to map out the factors postulated in the Health Belief Model.

#### Socio-Demographic Data

Besides acquiring information on gender (male/female/diverse), age (in years; retrospectively subsumed in 6 groups: 18–29/30–39/40–49/50–59/60–69/70 + year-old) and highest educational level (7 categories from “no graduation” to “university degree”) we asked participants for annual income (up to 25.000 €/25.000–40.000 €/40.000–70.000 €/70.000–100.000 €/> 100.000 €) and the number of people per household. Since in the HBM socio-demographic variables are postulated as essential moderating influencing factors, they have to be assessed and analyzed in our study in order to predict preventive behavior.

#### HBM Questionnaire

As most HBM-related studies have used self-report measures of behavior, we decided to do so as well. There are two ways in which the content of the items of the questionnaire can be determined. First, the literature can be searched for previous HBM studies using appropriate instruments to the objective at hand. For example, for breast self-examination the HBM questionnaire developed by Champion (32) has been widely used, for a more recent publication in this context see e.g., Norfariha (33). Furthermore, there are published HBM scales developed in the areas of health beliefs for antibiotic therapy (34) or related to cardiovascular disease (35) and sleep apnea (36). However, we could not identify appropriate, previously developed HBM measures, for our study. We thus decided to take the second option and develop our own instrument. The first step involved generating items that purport to measure HBM components. Previous HBM studies were used as a guide in this regard. Content (face) validity was ascertained by three experts (psychologists). We considered simultaneously measuring health beliefs and health behavior, although there may be social desirability biases (37). At the time the study was conducted, it was assumed that the pandemic would subside after the first lock-down. Conducting two consecutive studies building on one another did therefore not seem fruitful.

The result was a 46-item-version of the health belief questionnaire consisting of items designed to reflect the contents of the HBM:

*Perceived Severity* (2 items): “The coronavirus is harmless—dangerous” (5-point Likert scale) “The coronavirus is comparable to influenza—more dangerous than influenza” (5-level Likert scale);

*Perceived Susceptibility* (3 items on the dangerousness of the virus itself, the risk of becoming ill, and the risk of transmitting the infection), e.g., “To what extent are you susceptible of catching COVID-19?” (5-point Likert scale); The internal consistency (Cronbach's alpha) in our sample is α = 0.60.

*Perceived Barriers due to health-promoting measures* (10 items on negative feelings related to the behavioral measures), e.g., “Self-isolation due to coronavirus makes me angry” (5-point Likert scale); The internal consistency (Cronbach's alpha) in our sample is α = 0.87.

*Perceived Benefits of health-promoting measures* (15 items on the assessment of the value and efficacy of health-promoting behavioral measures: quarantine, circulation and traveling restrictions, smart working, cancellation of events), e.g., “Self-isolation due to coronavirus is reasonable” (5-level Likert scale), “Self-isolation due to coronavirus can prevent it from spreading” (5-point Likert scale); The internal consistency (Cronbach's alpha) in our sample is α = 0.90.

*Engagement in health-promoting behaviors* (16 items on engagement in health-promoting behaviors—keeping a safe distance, avoiding shaking hands, hugging, use of public transport, hand hygiene, mobile phone disinfection), e.g., “I have observed the following rules to protect myself from infection: Generally avoiding close proximity to other people” (dichotomy); The internal consistency (Cronbach's alpha) in our sample is α = 0.83.

Standardized scales to assess the emotional reaction to the pandemic-crisis and the premorbid vulnerability to intense stress reaction were used in the following order:

The German version of the *Positive and Negative Affect Schedule*—*PANAS* was adapted from the one developed by Watson et al. (38) to measure emotional states. It consists of twenty adjectives that describe different emotions and feelings. The two groups consisting of ten terms are accurate markers of either positive or negative affect. Subjects assess their intensity on a 5-point scale ranging from “not at all” to “extremely.” The intent is to indicate the range of emotions at a given moment or over a period of time, and their chronic or transient nature. This study takes into consideration emotions related to the corona pandemic. The internal consistencies (Cronbach's alpha) for both subscales are α > 0.84.

*Stress Coping Style Questionnaire*—*SVF 78* (39): This questionnaire evaluates the coping style and the processing patterns in stressful situations. It is composed of 13 subscales, each describing pre-defined reactions to stress in terms of time and situation-stable (stressor) personal characteristics: underestimation, guilt denial, diversion, compensatory satisfaction, situation control, reaction control, positive self-instruction, perceived social support, avoidance, escape tendency, perseveration, resignation, self-accusation. The internal consistencies (Cronbach's alpha) of the SVF subtests are between α = 0.77 and α = 0.94.

*Uncertainty intolerance Scale*—*UI-18* (40): This diagnostic scale uses 18 items to measure intolerance for uncertainty. It combines three subscales “reduced ability to act,” “burden,” and “vigilance” rated on a 5-point Likert scale. The internal consistency for the entire scale is α = 0.90.

*State Trait Anxiety Inventory*—*STAI* (41): is a standard tool in anxiety and stress research consisting of two subscales incorporating 20 items (4-points Likert scale) to differentiate anxiety as a state (state = temporary emotional condition varying in intensity over time and according to the situation) and anxiety as a trait (trait = a relatively consistent personality pattern). The internal consistency for both subscales is α = 0.90.

### Participants

During the survey validity period, there were 5,007 registered accesses via the dedicated link. 15.10% of the participants (*N* = 756) completed the first page, where participants were informed of the content of the study as well as on the data processing and were asked to give written consent to the participation in the study. Only about 5.89% (*N* = 294) of the participants abandoned the questionnaire after the second page, where socio-demographic data was collected. The overall dropout rate of 38.83% (*N* = 1,944) of participants, who did not complete the questionnaire, is acceptable (42). After plausibility check 38 of the 3,044 filled out data sets were excluded because participants clicked through. In the end, 3,006 data sets (693 from Germany and 2,313 from Austria) were included in the evaluation.

### Statistical Analysis

The Statistical Package for the Social Sciences Program (SPSS Version 24) was used for data input, processing, and statistical analyses. We used an alpha level of 0.05 for all statistical tests.

One-way analyses of variance (ANOVA), followed by pairwise *post-hoc*-Tests (Bonferroni), were conducted to compare the HBM scales *Perceived Susceptibility, Perceived Severity of COVID19, Perceived Benefits of health-promoting measures, Perceived Barriers due to health-promoting measures* and *Engagement in health-promoting behaviors* between age and income groups. Mann-Whitney-U test was performed to assess gender differences.

**Figure 1:**
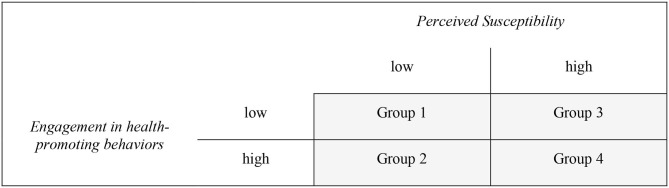
Groupclassification based on dichotomization of the scales *Perceived Susceptibility* (*M* = 8.72) and *Engagement in health-promoting behavior*s (*M* = 10.48).

Based on the Health Belief Model, four groups were formed according to the scales *Perceived Susceptibility* and *Engagement in health-promoting behaviors*, in which we performed dichotomization using the arithmetic mean of the two scales (Figure 1).

A multivariate analysis of variance (MANOVA), followed by a series of ANOVAs, were conducted to compare scores in the PANAS-, STAI-, SVF78-, UI-18*-*, and COVID-19-scales to explore differences within these four groups.

ANOVAs, followed by pairwise *post-hoc*-Tests (Bonferroni), were calculated on the socio-demographic variables *Age* and *Number of people per household* between the four groups, and Kruskal-Wallis test was performed to assess income differences.

## Results

### Demographics

The gender distribution shows a higher proportion of women participating in this study, demonstrated by 2,056 female respondents (68%), 928 male participants (31%), and 22 subjects (1%) who did not specify their gender. The average age was 35.3 years (*SD* = 11.7). The distribution of the socio-demographic variable “highest education level” also shows that the sample at hand consists of an above average educational level: 28.5% had a general qualification for university entrance, 59.5% a university degree. A total of 644 respondents declared living alone (21.4%), the rest cohabiting, for the most part in a two-person household (1,229 people or 40.9%). A quarter of the participants live with one or more children.

The participants who clicked through consisted of a higher proportion of woman (75%), the average age was 34.5 years. The distribution of the variable “highest education level” shows that 21% had a general qualification for university entrance, 60.5% a university degree.

### Descriptive Statistics and Socio-Demographic Analysis of the HBM-Questionnaire-Scores

Prior to conducting one-way and multivariate analyses of variance, the assumptions (homogeneity of the covariance matrixes, homogeneity of the variances, normality, and multicollinearity) were tested for all HBM and personality scales. The assumption of normality was not met for all scales (Shapiro-Wilk and Kolmogorov-Smirnov < 0.001), but MANOVA and one-way ANOVA tolerate violations to its normality assumption rather well [e.g., (43)]. The assumption of homogeneous covariance matrices was not given (Box-M-Test < 0.001). The homogeneity of variance assumption was considered satisfied for the scales *Burden due to Intolerance of Uncertainty, Positive Affect, Positive Stress Behavior* and *Negative Stress Behavior*, but eight of the twelve Levene's *F* tests were statistically significant (*p* > 0.05). Multicollinearity was not a confounding factor in the analysis (*r* < 0.90). Specifically, although the Levene's *F* test suggested that the variances associated with eight subscales were not homogenous, an examination of the standard deviations (see Table 1) revealed that the ANOVA would be robust in this case (45). Nevertheless, whenever Levene's test for homogeneity of variance was significant non-parametric statistics (Kruskal-Wallis) were used to confirm the effects obtained via the ANOVAs. In all cases, the Kruskal-Wallis tests confirmed the findings of the ANOVAs. Therefore, the results of the ANOVAs only were reported.

**Table 1:** Descriptive Statistics and ANOVAs results for personality scales and socio-demographic variables.

**Personality and socio-demographic measures**		**Group 1 (*n =* 450)**	**Group 2 (*n =* 984)**	**Group 3 (*n =* 467)**	**Group 4 (*n =* 1104)**	***p***	***F***	***η^2^***
STAI Trait Anxiety	*M*	38.40	39.43	41.71	41.94	<0.001	15.14	0.02
	*MD*	37.00	38.00	40.00	39.00			
	*SD*	10.50	11.52	11.59	11.96			
STAI State Anxiety	*M*	40.10	42.33	44.64	45.90	<0.001	32.16	0.03
	*MD*	38.00	40.00	43.00	45.00			
	*SD*	10.98	11.74	11.57	12.24			
PANAS Positive Affect	*M*	23.21	24.50	23.36	24.47	<0.001	8.98	0.01
	*MD*	23.00	24.00	23.00	24.00			
	*SD*	6.16	5.71	5.89	5.87			
PANAS Negative Affect	*M*	18.22	19.37	20.61	21.55	<0.001	38.74	0.04
	*MD*	17.00	18.00	20.00	21.00			
	*SD*	5.66	6.17	6.18	6.56			
UIA Reduced Ability to act due to	*M*	13.46	13.59	14.22	14.20	0.027	3.07	0.00
Intolerance of Uncertainty	*MD*	12.50	12.00	13.00	13.00			
	*SD*	5.54	6.02	6.03	6.17			
UIB Burden due to Intolerance of Uncertainty	*M*	16.24	16.68	17.22	17.59	<0.001	7.12	0.01
	*MD*	16.00	16.00	16.00	18.00			
	*SD*	5.62	5.94	6.11	6.16			
UIC Vigilance due to Intolerance of Uncertainty	*M*	16.73	17.22	17.05	17.50	0.080	2.26	0.00
	*MD*	16.00	17.00	17.00	18.00			
	*SD*	5.54	5.39	5.92	5.61			
SVF78 Positive Stress Behavior	*M*	18.53	19.21	18.45	19.00	<0.001	10.64	0.01
	*MD*	18.71	19.29	18.43	19.14			
	*SD*	2.90	2.80	2.88	2.84			
SVF78 Negative Stress Behavior	*M*	16.01	15.98	16.77	16.68	<0.001	6.14	0.01
	*MD*	15.50	15.75	16.75	16.50			
	*SD*	4.57	4.47	4.66	4.68			
Perceived Severity of COVID-19	*M*	7.42	8.15	7.75	8.33	<0.001	50.31	0.05
	*MD*	8.00	8.00	8.00	8.00			
	*SD*	1.71	1.36	1.54	1.39			
Perceived Benefits of	*M*	67.46	71.22	66.95	70.83	<0.001	68.26	0.06
health-promoting behavioral	*MD*	71.00	73.00	70.00	73.00			
measures	*SD*	9.80	5.30	8.94	5.21			
Perceived Barriers due to	*M*	14.05	13.51	15.12	14.57	<0.001	11.65	0.01
health-promoting behavioral	*MD*	12.00	12.00	13.00	13.00			
measures	*SD*	5.67	4.73	6.03	5.60			
Age	*M*	35.11	36.60	33.88	34.71	<0.001	7.31	0.01
	*MD*	32.00	34.00	32.00	32.00			
	*SD*	11.98	12.65	11.01	10.92			
Number of people per household	*M*	2.50	2.33	2.57	2.51	0.001	5.22	0.01
	*MD*	2.00	2.00	2.00	2.00			
	*SD*	1.30	1.16	1.38	1.36			

*Group 1 (157 males, 291 females) low Perceived Susceptibility, low Engagement in health-promoting behaviors; Group 2 (306 males, 671 females) low Perceived Susceptibility, high Engagement in health-promoting behaviors; Group 3 (151 males, 312 females) high Perceived Susceptibility, low Engagement in health-promoting behaviors; Group 4 (314 males, 782 females) high Perceived Susceptibility, high Engagement in health-promoting behaviors*.

*Effect sizes: η^2^ = 0.01 small, η^2^ = 0.10 medium, η^2^ = 0.25 large (44)*.

Health-promoting measures implemented by the Austrian and German governments (self-isolation, quarantine, travel restrictions, smart working, and event-cancellations) are considered to be highly effective. The *Perceived Benefits*' median value is 72 out of 75 (*M* = 69.85, *SD* = 7.01), revealing a high level of endorsement. One-way analyses of variance show significant differences between the different age groups [*F*_(5, 3, 000)_ = 2.91, *p* < 0.001 η^2^ = *0*.01] but not income groups [*F*_(4, 3, 001)_ = 1.16, *p* = 0.327, η^2^ = *0*.00]. *post-hoc*-Tests (Bonferroni) demonstrate that the group of 30–39 year-olds had the highest acceptance, the 50–59 year-olds the lowest. The Mann-Whitney-U test displays gender differences (*z* = −4.71, *p* < 0.001), with women ascribing more effectiveness to the measures than men. Besides the broad approval of the governments' action, the level of discomfort stemming from the restrictive measures (*Perceived Barriers*) to tackle the infection remains relatively low. A skewed distribution is observed, with a median of 12 out of 50 (*M* = 14.23, *SD* = 5.44). Nevertheless, this essentially positive picture does not apply to all groups to the same extent. One-way analyses of variance show different levels of emotional distress between age groups [*F*_(5, 3, 000)_ = 9.40, *p* < 0.001, η^2^ = 0.02] and income groups [*F*_(4, 3, 001)_ = 5.99, *p* < 0.001, η^2^ = 0.01]. As *post-hoc*-Tests (Bonferroni) illustrate, the group of 18–29-year-olds and the group with the lowest income were less tolerant to discomfort than all other groups.

As for the level of *Engagement in health-promoting behaviors* (*M* = 10.48, *SD* = 2.7), no significant differences are observed among different age groups [*F*_(5, 3, 000)_ = 2.04, *p* = 0.070, η^2^ = 0.00] and with regard to gender (*z* = −1.06, *p* = 0.291).

The level of *Perceived Susceptibility* (*M* = 8.72, *SD* = 2.36) shows significant differences among age groups [*F*_(5, 3, 000)_ = 7.42, *p* < 0.001, η^2^ = 0.01] and income groups [*F*_(4, 3, 001)_ = 3.45, *p* = 0.008, η^2^ = 01]. *post-hoc*-Tests (Bonferroni) demonstrate that the groups of 18–29-year-olds and 30–39-year-olds reported a higher subjective risk of disease than the 60–69-year-olds. The group with the lowest income shows a higher perceived susceptibility than all other groups.

### Group Comparison

**Figure 2:**
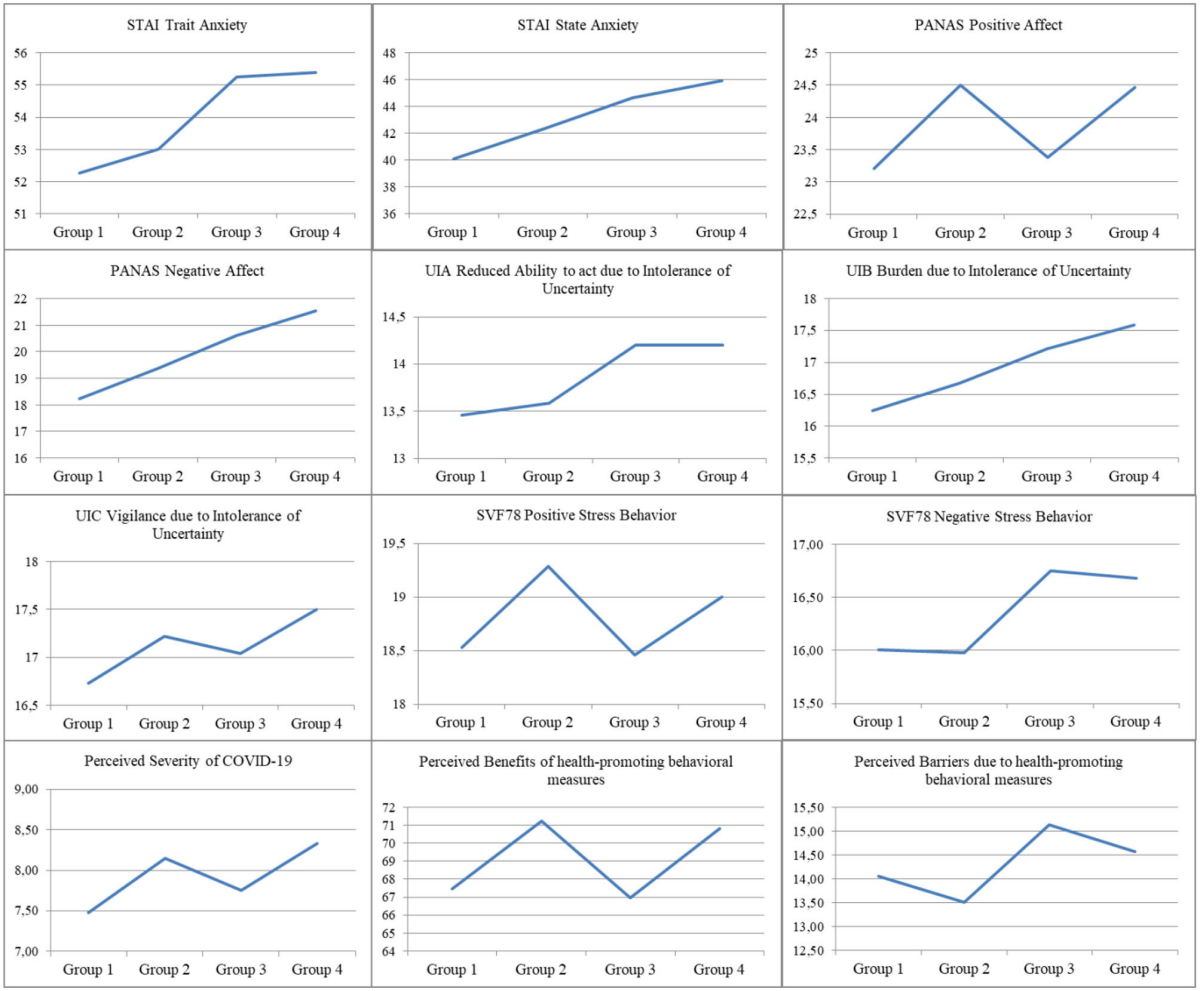
Mean values of all personality scales in the four groups.

The four groups created according to health-promoting behavior display significant differences in almost all personality dimensions derived from the personality questionnaires (see Table 1 and Figure 2). They differ in terms of *Perceived severity of COVID-19, Perceived benefits of health-promoting measures, Perceived barriers of health promoting measures, UIA, UIB, UIC, Positive Affect, Negative Affect, State Anxiety, Trait Anxiety, Positive Stress Behaviors*, and *Negative Stress Behaviors [*Λ = 0.86, *F*_(36, 8, 835)_ = 12.83, *p* < 0.001; η^2^ = *0.05*].

Regarding the subsequently conducted one-way ANOVAs, group differences can be identified regarding to the personality traits *Trait Anxiety* [*F*_(3, 3, 001)_ = 15.14, *p* < 0.001, η^2^ = 0.02], *State Anxiety* [*F*_(3, 3, 001)_ = 32.16, *p* < 0.001, η^2^ = 0.03], *Positive Affect* [*F*_(3, 3, 001)_ = 8.98, *p* < 0.001, η^2^ = 0.01], N*egative Affect* [*F*_(3, 3, 001)_ = 38.74, *p* < 0.001, η^2^ = 0.04], *Reduced Ability to act due to Intolerance of Uncertainty* [*F*_(3, 3, 001)_ = 3.07, *p* = 0.027, η^2^ = 0.00], *Burden due to Intolerance of Uncertainty* [*F*_(3, 3, 001)_ = 7.12, *p* < 0.001, η^2^ = 0.01], *Positive Stress Behavior [F*_(3, 3, 001)_ = 10.64, *p* < 0.001, η^2^ = 0.01], *Negative Stress Behavior [F*_(3, 3, 001)_ = 6.13, *p* < 0.001, η^2^ = 0.01], *Perceived Severity of COVID-19* [*F*_(3, 3, 001)_ = 50.31, *p* < 0.001, η^2^ = 0.05], *Perceived Benefits of health-promoting behavioral measures* [*F*_(3, 3, 001)_ = 68.26, *p* < 0.001, η^2^ = 0.06], and *Perceived Barriers of health-promoting behavioral measures* [*F*_(3, 3, 001)_ = 11.65, *p* < 0.001, η^2^ = 0.01].

The results of the *post-hoc*-Tests are shown in Table 2. The STAI scales show that Group 1 and Group 2 display significantly lower trait and state anxiety values than Group 3 and Group 4 [*Trait anxiety*: Group 1: 38.40 (10.50), Group 2: 39.43 (11.52), Group 3: 41.71 (11.58), Group 4: 41.94 (11.97); *State anxiety*: Group 1: 40.10 (10.98), Group 2: 42.33 (11.74), Group 3: 44.64 (11.57), Group 4: 45.90 (12.24)]. The state anxiety in Group 1 is also significantly lower in comparison to Group 2.

**Table 2:** Bonferroni *post-hoc* tests.

**Personality and demographic measures**	**Group**	**Group**	***p***
STAI Trait Anxiety	1	3	<0.001
	1	4	<0.001
	2	3	0.003
	2	4	<0.001
STAI State Anxiety	1	2	0.005
	1	3	<0.001
	1	4	<0.001
	2	3	0.003
	2	4	<0.001
PANAS Positive Affect	1	2	0.001
	1	4	0.001
	2	3	0.003
	3	4	0.003
PANAS Negative Affect	1	2	0.007
	1	3	<0.001
	1	4	<0.001
	2	3	0.003
	2	4	<0.001
	3	4	0.037
UIB Burden due to Intolerance	1	4	<0.001
of Uncertainty	2	4	0.003
SVF78 Positive Stress Behavior	1	2	<0.001
	1	4	0.019
	2	3	<0.001
	3	4	0.003
SVF78 Negative Stress Behavior	2	3	0.013
	2	4	0.003
Perceived Severity of COVID-19	1	2	<0.001
	1	3	0.004
	1	4	<0.001
	2	3	<0.001
	2	4	0.026
	3	4	<0.001
Perceived Benefits of health-promoting	1	2	<0.001
behavioral measures	1	4	<0.001
	2	3	<0.001
	3	4	<0.001
Perceived Barriers due to	1	3	0.016
health-promoting behavioral measures	2	3	<0.001
	2	4	<0.001
Age	2	3	<0.001
	2	4	0.001
Number of people per household	2	3	0.006
	2	4	0.008

*Only relevant results are included*.

*Group 1, low Perceived Susceptibility, low Engagement in health-promoting behaviors; Group 2, low Perceived Susceptibility, high Engagement in health-promoting behaviors; Group 3, high Perceived Susceptibility, low Engagement in health-promoting behaviors; Group 4, high Perceived Susceptibility, high Engagement in health-promoting behaviors*.

Groups 1 and 3 have a significantly lower positive affect and weaker positive stress processing strategies than Groups 2 and 4 [*PANAS Positive Affect:* Group 1: 23.21 (6.16), Group 2: 24.50 (5.71), Group 3: 23.38 (5.89), Group 4: 24.47 (5.87); *SVF78 Positive Stress Behavior:* Group 1: 18.53 (2.90), Group 2: 19.29 (2.80), Group 3: 18.46 (2.88), Group 4: 19.00 (2.84)]. Group 1 also has a distinctly lower negative affect than all other groups [*PANAS Negative Affect:* Group 1: 18.22 (5.66), Group 2: 19.37 (6.17), Group 3: 20.62 (6.18), Group 4: 21.55 (6.57)]. Group 2 also achieved significantly lower negative affect scores than Group 3 and Group 4 and registered noticeably less negative stress processing strategies than these two groups [*SVF78 Negative Stress Behavior*: Group 2: 15.98 (4.47), Group 3: 16.75 (4.67), Group 4: 16.68 (4.68)].

Additionally, COVID-19 is rated as significantly less dangerous by Group 1 than by all other groups, with Group 3 also registering significantly lower values in this section than Groups 2 and 4 [*Perceived Severity of COVID-19*: Group 1: 7.48 (1.71), Group 2: 8.15 (1.36), Group 3: 7.75 (1.54), Group 4: 8.33 (1.39)]. The benefits of health-promoting behavioral measures were rated significantly lower in groups 1 and 3 [*Benefits of health-promoting measures*: Group 1: 67.46 (9.80), Group 3: 66.97 (8.93)], than in groups 2 and 4 [Group 2: 71.22 (5.30), Group 4: 70.83 (52.08)]. The emotional distress due to the health-promoting behavioral measures is significantly lower in Group 1 compared to all other groups [*Perceived Barriers:* Group 1: 14.05 (6.67), Group 2: 13.51 (4.73), Group 3: 15.13 (6.02), Group 4: 14.57 (5.60)].

One-way analyses of variance show significant differences regarding the the socio-demographic variables *Age* [*F*_(3, 3, 001)_ = 7.31, *p* < 0.001, η^2^ = 0.01] und *Number of people per household* [*F*_(3, 3, 001)_ = 5.22, *p* = *0*.001, η^2^ = 0.01]. Group 2 had a higher average age than Group 3 and Group 4 [Group 1: 35.11 (11.98), Group 2: 36.60 (12.65), Group 3: 33.88 (11.01), Group 4: 34.71 (10.92)]. Furthermore, there was a significant difference between Group 2 [2 33 (1.16)] and Group 3 [2.57 (1.38)] as well as Group 2 and Group 4 [2.51 (1 36)] regarding the number of people per household. Group 2 had the smallest number, Group 3 the largest. The Kruskal-Wallis test shows no significant differences between the income groups (χ^2^ (6) = 5.86, *p* = 0.119).

## Discussion

### Interpretation of the Results

The Health Belief Model (HBM) classifies four groups or varying personality variables based on the perceived susceptibility and engagement in health-promoting behavioral measures:

**Group 1** (*N* = 450) shows a lower degree of perceived severity of coronavirus as well as a lower degree of perceived susceptibility to the disease. Subjects in this group indicate a poor threat perception and thus a weaker motivation to engage in health-related behaviors. The statutory preventative measures are considered relatively ineffective and disproportionate and are consequently not associated with potential benefits. This group shows a lower level of concern and uncertainty and ascribes only minor positive and negative affect to the pandemic. Emotional stress or discomfort due to perceived barriers remain contained, as the cost-benefit analysis results in reduced engagement in protective measures. Group 1, which constitutes the smallest group within the sample, mainly displays an underestimation of the pandemic.

Individuals belonging to **Group 2** (*N* = 984) report a lower perceived susceptibility but still adhere to the imposed measures. The estimated personal risk is not deemed alarming, but the virus is regarded as highly dangerous (perceived severity). The governments' measures are considered adequate (perceived benefits), but the resources available to deal with the crisis allow for less negative responses to perceived barriers. Levels of anxiety are not reported to be high, thus there is a rather positive affect related to the pandemic, as well as engagement in positive stress management strategies. This group demonstrates how positive personality variables can regulate health-related behavior. The sense of responsibility toward others motivates compliance with protective measures, along with a lack of internal resistance. Group 2, the second-largest within the sample, can be defined as “resource-conscious and responsible.”

**Group 3** (*N* = 468), the second smallest group, estimates the individual risk of infection as high, but only partially complies with protective measures as the perceived benefits do not represent an essential motivation. Indeed, the measures to tackle the virus are viewed as excessive, given the lower perceived severity of the disease. The HBM's cost-benefit balance reveals lower motivation in this group, as the response to the measures entail negative emotions (perceived barriers) and limited human resources as support throughout these challenging times. Subjects indicate increased anxiety, higher negative affect, and fewer positive stress processing strategies. This group also shows how personality variables indirectly influence health behavior. In this instance, the perception of a higher subjective risk is inversely proportional to the necessary resources to abide by protective measures without unreasonable personal sacrifice in terms of emotional discomfort and stress. This group is thus characterized by a minimized threat perception and an activation of complex fear defense mechanisms.

Individuals who belong to **Group 4** (*N* = 1,004) report both a higher perceived susceptibility and perceived severity. Therefore, according to the HBM, the subjective threat is assumed as an essential incentive. Participants in this group classify protective measures as adequate and reasonable and value the resulting perceived benefits. Due to the level of distress, processing the situation (perceived barriers) involves negative emotions. The lack of personal resources generates increased anxiety, generally higher affects (both negative and positive), and more negative stress processing strategies. Compared to the first three groups, this last one experiences insecurity as a burden, resulting in higher observance of protective measures despite the perceived barriers. The primary motivation is the perceived threat of the virus and the reliance on the effectiveness of the measures (perceived benefits). This group, making up about a third of the sample, can be labeled as “anxiously compliant.”

Overall—in line with the meta-analysis of the effectiveness of HMB variables in predicting behavior of Carpenter (46)—the benefits and barriers also turned out to be very important components of HBM in our group formation. On the other hand, we could not show—as Janz and Becker (47) have pointed out in their review—that barriers, benefits, and susceptibility were good predictors of behavior whereas severity was not. These early findings are not reflected in our data, because our findings showed that a low severity assessment goes hand in hand with lower engagement in health promoting behavior.

This group allocation shows—as Lippke und Renneberg (29) have noted and as has been shown in older (27) as well as more recent (28) reviews—that the factors postulated in the HBM for the realization of health behavior are influenced by further variables, and that the HBM therefore has to be expanded. In this study, we could demonstrate that at least with regards to the compliance with protective measures in pandemic crises, psychological variables (e.g., coping strategies and fear) play a crucial role. It becomes apparent that premorbid anxiety in this major crisis contributes to an increased fear and stress reaction, and in addition, coping strategies as essential resources are often poorly developed. A lack of coping skills contributes to strong negative emotions due to the preventive measures, which in turn influence the cost-benefit calculation postulated in HBM. Despite the lack of a comparable study connecting the concept of HBM with psychological aspects in pandemics, other studies do show similar moderating personality variables in the processing of lockdown associated stresses [e.g., (48)].

### Practical Implications: Group-Oriented Support Measures

Which measures can increase compliance with the statutory preventative measures within groups showing little acceptance? What can be done to support groups with limited resources to deal with a pandemic?

As for **Group 1**, the issue of underestimation requires promoting compliance with health behaviors. While reporting few negative emotions and uncertainties, this group runs the risk of maladaptive psychological strategies to safeguard health. It is expected that the “indifference” that characterizes Group 1 is by no means a positive psychological strategy, but rather an expression of helplessness and impotence connected to the corona crisis. The connection between emotional numbness as a reaction to experienced powerlessness stems from psychotraumatology (49). Additionally, it has to be noted that the survey was conducted at an early stage of the pandemic, when COVID-19 had not fully impacted the respondents' closest relationships, and information was still contradictory. Acting on multiple levels is paramount. The individual must perceive that the target behavior will provide strong positive benefits [e.g., (50)], providing accurate information on efficient protection measures and the threat's real extent is therefore of great importance. It is also essential to increase the sense of responsibility and empathy in this group. The fact that empathy is an important factor in complying with certain measures was previously demonstrated by Kim and Cooke (51) in their application of HBM to the ecological protection of the ocean.

**Group 2** (resource-conscious and responsible) benefits from resource-conscious and responsible behavior. This coping mechanism represents an exemplary behavior in fostering action in the interest of society. In line with the theory of reasoned action (52), they can serve as positive role models for Group 1 and increase social pressure to comply with the prevention measures.

People in **Group 3** are exposed to an increased risk of contagion. As precautionary measures are less observed, self-care needs to be promoted, potential further steps as well as beneficial responses need to be clarified, in order to avoid underestimation of the virus. As with Group 4, psychological help is essential to reduce anxiety and cope with the additional burdens as a result of the health-promoting. measures.

**Group 4** (anxiously compliant), like Group 3, tends to respond to the disease with stress and concern. However, unlike Group 3, Group 4 observes health-related protective measures. The appropriate strategy involves providing health services such as general medical checkups and counseling (telemedicine). Therefore, it is of the utmost importance to train health professionals in assisting those who might not be seeking medical help out of fear of COVID-19. Physicians and therapists might use telehealth as a valid option and notify their patients about the precautions taken in their practice (53, 54). The disproportionate fear reaction is related to uncertainties generated by the novel corona crisis and inconsistent information and conspiracy theories spread by the media.

In general, and particularly for Groups 3 and 4, prevention should tackle the stigma around psychological responses such as anger, excessive demands, stress, fear, and feelings of helplessness. Furthermore, it is fundamental to educate people about the delayed effects of psychological stress (1) to foster a better understanding and encourage recourse to psychosocial assistants.

### Methodological Limitations

Constraints of generalizability. The strongest limitations of this study result from online recruitment (forums and social media platforms) creating a snowball sampling procedure. This non-probability sampling technique may reflect a bias in the self-selection of the participants whose central characteristics do not correspond to the overall German and Austrian population. This restricts a potential replication of the study (55). The sample in this study, has, for example, a higher proportion of women with a high level of education. Furthermore, the data of the non-completers was analyzed to a limited extent.

Constraints of self-assessments. At the same time, as data derived from self-assessments does not constitute an accurate representation of behaviors, statements about the actual compliance with preventive measures require caution. However, since the survey was anonymous, we can presume a low social desirability tendency, although recall biases may influence self-assessments [see retrospection effect, e.g., (56)].

Constraints of response tendencies. In two scales (*Perceived Severity* of COVID-19, *Perceived Benefits of health-promoting measures)* there is a problem with range restriction as most respondents selected the highest response category. A nuanced assessment of the measured construct has thus not been possible.

### Prospect of Future Research

Objective data collection strategies are fundamental in representative surveys. It is advisable to repeat the survey in a post-acute phase of the pandemic, still requiring compliance with restrictions, for a follow-up of the samples' differences and characteristics. The same applies to comparative studies of the worst-affected countries. As the information supporting the conclusions of this study indicates that personality traits can indeed shape health behaviors, further research to expand the HBM should concentrate on factors such as anxiety and stress processing styles. Likewise, it should be evaluated whether the group-specific support measures derived from the results of the study are effective and tested whether the processing mechanisms known from trauma research are consistent in pandemic stress situations as assumed for Group 1.

## Data Availability Statement

The raw data supporting the conclusions of this article will be made available by the authors, without undue reservation.

## Ethics Statement

The studies involving human participants were reviewed and approved by Ethics Committe of the Sigmund Freud University Vienna. The patients/participants provided their written informed consent to participate in this study.

## Author Contributions

CE: study design, development of the questionnaire, data collection, interpretation, and writing the manuscript. MG and JA: data evaluation. LH: development of the questionnaire and data collection. SK: supervision of the study. SH-B: data evaluation, interpretation, and writing the manuscript. All authors contributed to the article and approved the submitted version.

## Conflict of Interest

The authors declare that the research was conducted in the absence of any commercial or financial relationships that could be construed as a potential conflict of interest.
