# Supplementary material for: The Relationship Between the Implementation of Statutory Preventative Measures, Perceived Susceptibility of COVID-19, and Personality Traits in the Initial Stage of Corona-Related Lockdown: A German and Austrian Population Online Survey
Source: Front Psychiatry. 2021 Jan 27;12:596281. doi: 10.3389/fpsyt.2021.596281 (PMC7873476; doi:10.3389/fpsyt.2021.596281)
Supplement: Supplementary file 1 [file Data_Sheet_1.docx]

**APPENDIX**

1. **Translation of the MHB questionnaire scales**

*Perceived Severity* (2 items):

Please give your opinion on the following scales:

The coronavirus is…

| harmless | O | O | O | O | O | dangerous |
| --- | --- | --- | --- | --- | --- | --- |
| comparable to influenza | O | O | O | O | O | more dangerous than influenza |

*Perceived Susceptibility* (3 items):

| To what extent you are susceptible of catching COVID-19? | | | | | | |
| --- | --- | --- | --- | --- | --- | --- |
| not at all | O | O | O | O | O | great extent |
| To what extent you believe that you are able to avoid an infection with COVID-19? | | | | | | |
| not at all | O | O | O | O | O | great extent |
| To what extent you believe that you are a risk factor for transmitting the infection? | | | | | | |
| not at all | O | O | O | O | O | great extent |

*Perceived Barriers due to health-promoting measures* (10 items):

Please give your opinion on these measures:

| Self-isolation due to coronavirus… | | | | | | |
| --- | --- | --- | --- | --- | --- | --- |
|  | not at all |  | partly |  | a lot |  |
| makes me angry | O | O | O | O | O |  |
| frightens me | O | O | O | O | O |  |
|  |  |  |  |  |  |  |
| Quarantine due to coronavirus… | | | | | | |
| makes me angry | O | O | O | O | O |  |
| frightens me | O | O | O | O | O |  |
|  |  |  |  |  |  |  |
| Traveling restrictions due to coronavirus… | | | | | | |
|  | not at all |  | partly |  | a lot |  |
| makes me angry | O | O | O | O | O |  |
| frightens me | O | O | O | O | O |  |

| Smart working due to coronavirus… | | | | | | |
| --- | --- | --- | --- | --- | --- | --- |
| makes me angry | O | O | O | O | O |  |
| frightens me | O | O | O | O | O |  |

| Cancellation of events (exhibitions, sports, concerts, cinema) due to coronavirus… | | | | | | |
| --- | --- | --- | --- | --- | --- | --- |
| makes me angry | O | O | O | O | O |  |
| frightens me | O | O | O | O | O |  |

*Perceived Benefits of health-promoting measures* (15 items):

Please give your opinion on these measures:

| Self-isolation due to coronavirus… | | | | | | |
| --- | --- | --- | --- | --- | --- | --- |
|  | not at all |  | partly |  | a lot |  |
| is reasonable | O | O | O | O | O |  |
| can prevent spread | O | O | O | O | O |  |
| is exaggerated | O | O | O | O | O |  |
|  |  |  |  |  |  |  |
| Quarantine due to coronavirus… | | | | | | |
|  | not at all |  | partly |  | a lot |  |
| is reasonable | O | O | O | O | O |  |
| can prevent spread | O | O | O | O | O |  |
| is exaggerated | O | O | O | O | O |  |
|  |  |  |  |  |  |  |

| Traveling restrictions due to coronavirus… | | | | | | |
| --- | --- | --- | --- | --- | --- | --- |
|  | not at all |  | partly |  | a lot |  |
| is reasonable | O | O | O | O | O |  |
| can prevent spread | O | O | O | O | O |  |
| is exaggerated | O | O | O | O | O |  |
| Smart working due to coronavirus… | | | | | | |
|  | not at all |  | partly |  | a lot |  |
| is reasonable | O | O | O | O | O |  |
| can prevent spread | O | O | O | O | O |  |
| is exaggerated | O | O | O | O | O |  |

| Cancellation of events (exhibitions, sports, concerts, cinema) due to coronavirus… | | | | | | |
| --- | --- | --- | --- | --- | --- | --- |
|  | not at all |  | partly |  | a lot |  |
| is reasonable | O | O | O | O | O |  |
| can prevent spread | O | O | O | O | O |  |
| is exaggerated | O | O | O | O | O |  |

*Engagement in health-promoting behaviors* (16 items):

Due to the coronavirus, I have taken the following measures to protect myself from infection…

|  | yes | no |
| --- | --- | --- |
| generally avoid other people | O | O |
| avoid other people, who sneeze or cough | O | O |
| avoid of places visited by many people | O | O |
| keep a distance of two meters from other people | O | O |
| avoid public transport | O | O |
| avoid workplace | O | O |
| avoid flights | O | O |
| wash hands more often | O | O |
| use disinfectants | O | O |
| wear a protective mask | O | O |
| wear gloves | O | O |
| disinfect computer, mobile phone, etc. | O | O |
| avoid shaking hands | O | O |
| avoid hugs | O | O |
| avoid kisses | O | O |
| avoid sexuality | O | O |

1. **Verification of factor structure using explorative factor analysis (EFA)**

For the investigation, four scales, of the self-created questionnaire, were formed and checked by means of EFA (Principal Component Analysis [PCA] with a Varimax rotation). Items that were not specific enough or not sufficiently answered were excluded prior to the calculation as well as the scaling. For all scales the requirements for the EFA (Kaiser-Meyer-Olkin, Anti-Image Matrix, Bartlett’s test for sphericity, Cronbachs α) were checked. In the resulting factor solutions, items with charges per factor < .5 were suppressed. For the factorization the factors were used which resulted after the buckling criterion (Scree Plot), as well as eigenvalues > 1.

In regards to the prerequisites for conducting an EFA on the scale *Perceived Benefits of health-promoting measures* (15 items on individual's assessment of the value and efficacy of health-promoting behavioral measures: Quarantine, circulation and traveling restrictions, smart working, cancellation of events), the following results in regards to sampling adequacy can be considered as very good, with KMO= .89 and the Bartlett’s test of sphericity χ^2^ (105)= 25095.213, p< .001 show enough intercorrelations among variables. The Scree Plot suggested a two-factor solution, and showed two factors with an eigenvalue > 1, which declared 59% of total variance. The first factor (α = .90) represents the benefit that all different measures can *prevent Covid19* *from spreading* (e. g. “Self-isolation from the coronavirus can prevent it from spreading”), the second factor (α = .88) shows that the measures are *reasonable* and not exaggerated (e.g. “Self-isolation due to coronavirus is reasonable”).

The prerequisites for performing an EFA on the scale *Perceived Barriers due to health-promoting measures* (10 items on negative feelings related to the behavioral measures) result in the following values regarding sampling adequacy and can be regarded as very good, with KMO= .82 and the Bartlett’s test of sphericity χ^2^ (45)=13702.140, p< .001 show enough intercorrelations among variables. The Scree Plot suggested a two-factor solution, and showed two factors with an eigenvalue > 1, which explained 59% of total variance. The first factor (α = .82) reflects *fear* due to the measures taken by law (e.g. “Self-isolation due to coronavirus frightens me”), the second factor (α = .83) shows *anger* (e.g. “Self-isolation due to coronavirus makes me angry*”*).

Regarding the prerequisites for executing an EFA on the scale *Engagement in* *health-promoting behaviors* (16 items on engagement in health-promoting behaviors - keeping a safe distance, avoiding shaking hands, hugging, public transport, hands hygiene, mobile phone disinfection,…) the following results in regard to sampling adequacy can be considered as very good, with KMO= .87 and the Bartlett test χ^2^ (120)=15019.964, p< .001 indicates enough intercorrelations among variables. The Scree Plot suggested a four-factor solution, and showed two factors with an eigenvalue > 1, which explained 58% of total variance. The first factor (α= .81) reflects the general *avoidance of places and people* (e.g. workplace, public transport, flights, ...), the second factor (α= .73) forms *protective measures* (e.g. wear gloves, masks, disinfection, ...), the third factor (α= .74) shows *avoidance of physical contact* (e.g. avoid shaking hands, avoid hugs, wash hands often) and the fourth factor (α= .71) shows *avoidance of intimacy and sexuality* (e.g. avoid kissing, avoid sexuality).

The prerequisites for conducting an EFA on the scale *Perceived Susceptibility* (3 items on the dangerousness of the virus itself, the risk of becoming ill, and the risk of transmitting the infection), demonstrate the following results in regard to sampling adequacy and can be considered as good, with KMO= .59 and the Bartlett test χ^2^(3)= 1070.063, p< .001 indicates sufficient intercorrelations among variables. The Scree Plot suggested a one-factor solution, and showed one factor with an eigenvalue > 1, which explained 56% of total variance (α= .60).

1. **Intercorrelation matrix**

Intercorrelation matrix of all scales

| Scales | *Perceived Severity* | *Perceived Susceptibility* | *Perceived Barriers* | *Perceived Benefits* | *PANAS Positive affect* | *PANAS Negative affect* | *SVF78 Positive stress behavior* | *SFV78 Negative stress behavior* | *STAI*  *state anxiety* | *STAI*  *trait anxiety* |
| --- | --- | --- | --- | --- | --- | --- | --- | --- | --- | --- |
| *Perceived Severity* | 1 | 0.083 | 0.376 | -0.079 | 0.067 | 0.144 | 0.035 | 0.021 | 0.119 | 0.040 |
| *Perceived Susceptibility* | 0.083 | 1 | -0.037 | 0.137 | -0.015 | 0.221 | -0.029 | 0.096 | 0.205 | 0.151 |
| *Perceived Barriers* | 0.376 | -0.037 | 1 | -0.257 | 0.123 | 0.004 | 0.067 | 0.040 | -0.016 | -0.027 |
| *Perceived Benefits* | -0.079 | 0.137 | -0.257 | 1 | -0.189 | 0.566 | -0.053 | 0.292 | 0.439 | 0.394 |
| *PANAS Positive affect* | 0.067 | -0.015 | 0.123 | -0.189 | 1 | -0.228 | 0.330 | -0.239 | -0.375 | -0.365 |
| *PANAS Negative affect* | 0.144 | 0.221 | 0.004 | 0.566 | -0.228 | 1 | -0.089 | 0.486 | 0.710 | 0.590 |
| *SVF78 Positive stress behavior* | 0.035 | -0.029 | 0.067 | -0.053 | 0.330 | -0.089 | 1 | -0.243 | -0.242 | -0.329 |
| *SVF78 Negative stress behavior* | 0.021 | 0.096 | 0.040 | 0.292 | -0.239 | 0.486 | -0.243 | 1 | 0.525 | 0.690 |
| *STAI State anxiety* | 0.119 | 0.205 | -0.016 | 0.439 | -0.375 | 0.710 | -0.242 | 0.525 | 1 | 0.796 |
| *STAI Trait anxiety* | 0.040 | 0.151 | -0.027 | 0.394 | -0.365 | 0.590 | -0.329 | 0.690 | 0.796 | 1 |
